# ProAKAP4 as Novel Molecular Marker of Sperm Quality in Ram: An Integrative Study in Fresh, Cooled and Cryopreserved Sperm

**DOI:** 10.3390/biom10071046

**Published:** 2020-07-14

**Authors:** Marta F. Riesco, Luis Anel-Lopez, Marta Neila-Montero, Cristina Palacin-Martinez, Rafael Montes-Garrido, Mercedes Alvarez, Paulino de Paz, Luis Anel

**Affiliations:** 1 Itra-ULE, INDEGSAL, University of León, 24071 León, Spain; mferrs@unileon.es (M.F.R.); mneim@unileon.es (M.N.-M.); c.palacin@unileon.es (C.P.-M.); rmong@unileon.es (R.M.-G.); mmalvg@unileon.es (M.A.); ppazc@unileon.es (P.d.P.); laner@unileon.es (L.A.); 2 Cellular Biology, Department of Molecular Biology, University of León, 24071 León, Spain; 3 Anatomy, Department of Veterinary Medicine, Surgery and Anatomy, University of León, 24071 León, Spain; 4 Animal Reproduction and Obstetrics, Department of Veterinary Medicine, Surgery and Anatomy, University of León, 24071 León, Spain

**Keywords:** cooling, cryopreservation, rams, sperm quality, redox balance, ProAKAP4

## Abstract

To improve artificial insemination protocols in ovine species it is crucial to optimize sperm quality evaluation after preservation technologies. Emerging technologies based on novel biomolecules and related to redox balance and proteins involved in sperm motility such as ProAKAP4 could be successfully applied in ram sperm evaluation. In this work, a multiparametric analysis of fresh, cooled, and cryopreserved ram sperm was performed at different complexity levels. Samples were evaluated in terms of motility (total motility, progressive motility, and curvilinear velocity), viability, apoptosis, content of reactive oxygen species, oxidation‒reduction potential, and ProAKAP4 expression and concentration. As expected, cryopreserved samples showed a significant decrease of sperm quality (*p* < 0.05), evidencing different freezability classes among samples that were detected by ProAKAP4 analyses. However, in cooled sperm no differences were found concerning motility, viability, apoptosis, ROS content, and redox balance compared to fresh sperm that could explain the reported decrease in fertility rates. However, although the proportion of sperm ProAKAP4 positive-cells remained unaltered in cooled sperm compared to fresh control, the concentration of this protein significantly decreased (*p* < 0.05) in cooled samples. This altered protein level could contribute to the decrease in fertility rates of cooled samples detected by some authors. More importantly, ProAKAP4 can be established as a promising diagnostic parameter of sperm quality allowing us to optimize sperm conservation protocols and finally improve artificial insemination in ovine species.

## 1. Introduction

Artificial insemination (AI) is an essential and well-established tool in genetic improvement programs and mammalian breeds’ conservation. However, in ovine species this technique represents a significant constraint for genetic improvement programs [1,2,3,4] due to the short life span of fresh semen, together with the natural limitation on the number of semen doses that can be obtained per male [5]. Cooling and cryopreservation methods could solve the problem of sperm management in AI programs. Considerable efforts have been directed towards developing techniques for AI using frozen or cooled ram semen [5,6,7]. However, the success of ram sperm storage by cooling and freezing has been limited due to the lower resistance of ovine semen to thermal stress compared with other species [4,8,9]. Current sperm preservation methods compromise spermatozoa colonization and migration through the cervix, affecting fertility rates [4,5,10]. Therefore, it is necessary to perform detailed studies in order to optimize the existing protocols. In this sense, some bottlenecks concerning sperm quality analyses have been identified in this species. Traditional sperm quality analyses have limited prognostic value for the reproductive success of specific sperm doses [11,12]. Only the combination of several analyses has led to better fertility prediction [11]. A major reason for these variations and the lack of correlation is the fact that the ejaculate and the AI doses are composed of a diverse sperm population [13,14]. This suggests that spermatozoa from different males exhibit significantly different responses to the same freezing treatment. This is one important problem for standardizing sperm cryopreservation protocols in some species [15,16].

New approaches based on multiparametric analyses by flow cytometry have been proposed during the last decade as robust technologies to analyze sperm for in vitro fertilization programs in different mammalian species such as humans [17,18], stallions [19,20,21], and boar [22,23]. This technique enables the assessment of multiple attributes from individual sperm cells at the same time, and allows us to detect physiological heterogeneity within ejaculate in terms of cell subpopulations in a short period of time with high accuracy and reproducibility [24,25]. Redox balance plays a major role in controlling sperm functionality, and recent studies point out that redox regulation may be responsible for male subfertility or they offer alternatives to improve traditional methods of sperm conservation [26,27,28]. Although reactive oxygen species (ROS) are necessary for the normal physiological functioning of sperm, excessive oxidative stress can trigger apoptosis, leading to caspase-mediated destruction of DNA damage [27,29,30]. Nevertheless, recent research has suggested that the role of redox balance could be species-specific. In the case of equine sperm, a positive relationship between sperm oxidative stress and functionality has been observed, with implications for fertility [28]. It is widely known that sperm conservation methods (cryopreservation and cooling) provoke different levels of damage including an increase in plasma membrane fluidity, reduction of acrosome integrity, impairment of mitochondrial membrane potential, and finally a high production of ROS. The imbalance between ROS levels and physiologic antioxidant level can trigger oxidative stress responses [28]. New approaches applied to human and stallion sperm, based on the measure of oxidation‒reduction potential, have emerged [31,32]. RedoxSYS system is a novel technology based on a galvanostatic measure of electrons that presents static oxidation reduction potential ORP (sORP) and capacity oxidation reduction potential ORP (cORP) measures, with static referring to the passive or current state of activity between oxidants and antioxidants [33,34]. Consequently, the redox balance should be studied in an integrative manner, in combination with other sperm quality markers such as membrane integrity or apoptosis occurrence, to gain more predictive values on fertilization success [35]. The obtained results could be enlightening to optimize some cooling and freezing protocols with IA purposes. During conservation procedures, seminal plasma and sperm proteins modulate sperm survival and functionality determining its freezability (resilience to withstand cryopreservation). Specific biomolecules could act as freezability biomarkers providing new insights into the mechanisms underlying sperm cryodamage. Some of these proteins have been recently characterized in ruminants presenting different roles on sperm functionality: stress related proteins (glutathione s-transferase mu 5 [36], GSMT5, superoxide dismutase [37], SOD, and reduced glutathione [37], GSH), ion transportation (voltage-dependent anion-selective channel protein 2 [36], VDAC2), sperm motility (ATP synthase subunit beta [36], ATPB1), acrosome reaction (such as Phospholipase A2, PLA2 [38]), chaperone proteins (heat-shock proteins, HSPs, specifically HSP90 [39,40] and HPSPA8 [41]) carbonylated proteins related to oxidative modifications [42], lipid peroxidation (acidic seminal fluid protein [43], aSFP) capacitating proteins [44] (14 proteins related to mitochondrial activity, sperm motility, oocyte recognition, signaling, spermatogenesis, and the apoptosis-stress response), among others [44,45].

Emerging technologies based on biomolecules capable of predicting fertilization success and pinpointing the causative agents such as oxidative stress that can be targeted to prevent reproductive failure have recently been characterized [26]. A novel sperm protein and its precursor, AKAP4 and proAKAP4, respectively, have been previously established as molecular markers of sperm quality in different species (mice [46], stallions [47], boar [48], and humans [49,50]) due to their high correlation with sperm motility. AKAP4 and proAKAP4 are the most abundantly expressed proteins of the sperm fibrous sheath in all mammals, being part of the principle piece of the flagellum [51,52,53]. Moreover, the proAKAP4 precursor molecule has recently emerged as a fertility indicator in a number of species such as humans, boar, and bulls [47,48,50,54,55,56,57,58] providing it with an added value as a diagnostic biomarker of overall semen quality.

In the present study, we performed an integrative analysis of ram sperm quality in fresh, cooled, and cryopreserved samples, combining traditional analysis (motility and kinetic parameters), multiparametric assays by flow cytometry (viability, apoptosis and ROS content), and novel approaches based on redox balance and ProAKAP4 detection to improve sperm quality analyses in this species. These new approaches could significantly improve ram sperm quality evaluation after cooling and freezing technologies in order to optimize the sperm conservation protocols to enhance AI technologies in ovine species.

## 2. Materials and Methods

### 2.1. Ethics Statement

The current study was performed according to the Guidelines of the European Union Council (86/609/EU, modified by 2010/62/EU), following Spanish regulations (RD/1201/2005, abrogated by RD/2013) for the use of laboratory animals. All experimental protocols and procedures were approved by the institutional Animal Care and Use Committee at the University of León (Spain) (ÉTICA-ULE-013-2018).

### 2.2. Sample Collection

Eight adult Assaf rams of proven fertility (mean ± S.E.M.: 41.8% ± 3.1, range: 27.2–60.0%) aged between five and seven years were the subjects of the experiments. Animals were housed and fed with a standard balanced diet at the Animal Selection and Reproduction Center of the Junta de Castilla y León (CENSYRA) (Villaquilambre, León, Spain). Moreover, adequate and clean water was provided. Regular veterinarian inspections were performed with special attention to nutrition, as well as general and reproductive disease control.

Ram ejaculates were (one per male) obtained from trained males (weekly semen collections, twice a week) during the breeding season (autumn). Ejaculate was collected by artificial vagina (water at 40 °C) and the tubes were kept in a water bath at 35 °C during the initial evaluation of semen quality. Volume was measured and determined with a graduated tube of polystyrene. Mass motility was assessed with a subjective score of 0‒5 by a microscope equipped with a warmed stage programmed at 37 °C using a 10× objective (Leica DM LB, Meyer Instruments, Houston, USA). Sperm concentration was analyzed by a cell counter (NucleoCounter SP-100, ChemoMetec, Allerod, Denmark). Only ejaculates of good quality were used (volume ≥ 0.5 mL, mass motility ≥ 3, and concentration ≥ 3000 × 10^6^ sperm/mL).

### 2.3. Experimental Groups and Sample Treatments

Each ejaculate was split into three subsamples to obtain the experimental groups: fresh, cooled, and cryopreserved sperm. Fresh samples were diluted with the same volume (1:1) of TES- Tris- fructose media (*N*-Tris(hydroxymethyl)methyl-2-aminoethanesulfonic acid, TES 224 mM, Tris 85 mM, fructose 13 mM, adding 20% clarified egg yolk) (TTFM) and were analyzed immediately. Sperm cooling at 15 °C for 6 h and freezing-thawing protocols were carried out in the standard conditions previously published by our group for this species [10,59]. Briefly, in cooled samples, semen was diluted down to 1600 × 10^6^ of spermatozoa/mL in INRA96 medium^®^ (IMV Tecnhologies, L’Aigle, France), packed in 0.25-mL French straws and stored at 15 °C for 6 h. In cryopreserved samples, the previously diluted sperm (1:1) in TTFM was subject to slow cooling (−0.25 °C/min) until 5 °C using a programmable water bath and then frozen using a programmable biofreezer (Kryo 10 Series III; Planer PLC, Sunbury-on-Thames, UK) at a rate of −20 °C/min down to −100 °C. Then, the straws (100 × 10^6^ of spermatozoa/mL) were kept in liquid nitrogen containers for at least a week, and thawing was carried out in a water bath at 65 °C for 6 s. A sperm washing step with PBS (1mL) and centrifugation (600× *g* for 10 min) were included before sperm analyses in each experimental group (fresh, cooled, and cryopreserved).

### 2.4. Sperm Motility and Kinetic Parameters

Sperm motility, kinetics, and concentration assessments were performed using computer assisted sperm analysis (Sperm Class Analyzer (SCA) 6.3.0.59, Microptic S.L., Madrid, Spain) set to capture at 100 frames/second. Sperm samples of the three experimental groups (fresh, cooled, and cryopreserved) were diluted to a final concentration of 2 × 10^6^ sperm cells/mL in PBS (pH = 7.5, 300 mOsm/kg) and warmed to 37 °C on a warmed plate. Five microliters of the diluted semen of each experimental group was dropped onto a Makler counting chamber (10 µm depth; Sefi Medical Instruments, Mumbai, India) and analyzed with SCA software v. 6.3.0.59 (Microptic, Barcelona, Spain). The SCA system consisted of an optical phase-contrast Nikon Eclipse microscope (Nikon, Tokyo Japan) equipped with a Basler A312fc digital camera (Basler Vision Technologies, Ahrensburg, Germany) with a warmed stage (37 °C) using a 10× objective with negative phase contrast specifically set for ram spermatozoa (1 µm^2^ < particle area < 20 µm^2^; sperm characterization following curvilinear velocity (VCL): > 15 μm/s. At least 400 sperm cells from four different randomly selected fields were captured and analyzed. The sperm quality parameters included in our study were: the percentage of total motile spermatozoa (TM, %), progressive motility (PM, %), and a standard kinematic parameter: curvilinear velocity (VCL, μm/s). A total of eight individual males were analyzed (1 ejaculate per male) including the same males in each experimental group.

### 2.5. Multiparametric Flow Cytometry Analyses

Sperm samples of different experimental groups were diluted in PBS medium to obtain a total of 2 × 10^6^ of spermatozoa per sample; these samples were washed and centrifuged at 500× *g* for 10 min at RT. Lyophilized Zombie Violet™ dye (Biolegend, Madrid, Spain) was reconstituted in dimethyl sulfoxide (DMSO) following the manufacturer’s instructions (100 µL of DMSO to one vial of Zombie Violet™ dye). CellEvent™ Caspase-3/7 (Thermo Fisher, Madrid, Spain) and CellROX™ Deep Red (Thermo Fisher, Madrid, Spain) were purchased as a 2 mM and 2.5 mM stabilized solution, respectively. Stock solutions of fluorescence probes were prepared at 1 µL and kept at −20 °C in the dark until needed.

Zombie Violet™ stock solutions were resuspended in 1 mL of PBS while CellEvent™ Caspase- 3/7 and CellROX™ in 10 μL. The supernatant was discarded, and the sperm pellet was incubated at RT for 30 min in the dark with 96 μL of Zombie Violet™ (membrane integrity probe) (1:1000 final dilution, Biolegend, Madrid, Spain), 2 μL of CellEvent™ Caspase-3/7 (apoptosis marker) (4 µM final concentration, Thermo Fisher, Madrid, Spain), and 2 μL of CellROX^®^ (ROS content labeling) (5 μM final concentration, Thermo Fisher, Madrid, Spain). After that, another washing step was performed to stop cell staining, and the pellet was resuspended in 1 mL of PBS, carrying out the analysis immediately by flow cytometry (Supplementary Figure S1a–c). For this assay, 5‒8 individual males were analyzed in each experimental group.

For proAKAP4 labelling, after a sperm washing step with PBS, a total of 5 × 10^6^ sperm cells per sample were fixed in paraformaldehyde at 3% (*v*/*v*) in PBS (Merck, Spain) for 15–20 min at RT in the dark. After centrifugation at 600× *g* for 10 min, spermatozoa were washed twice in 1 mL of PBS and, after the last centrifugation, the spermatozoa pellet was resuspended in 100 µL of PBS. Sperm cells were permeabilized with 0.2% (*v*/*v*) Triton X-100 (Merck, Spain) for 1 h at RT. Then, after two washing steps with PBS, samples were blocked with 3% (*v*/*v*) BSA/PBS for 1 h at RT. After that, primary antibody anti-proAKAP4 (clone 6F12) (4BioDx, 4BDX-1701, Lille, France) was added and diluted in PBS with 0.3% Triton X-100 to a final dilution of 1 µg/mL. Spermatozoa were incubated at 4 °C overnight. Spermatozoa were washed twice with 1 mL of PBS at RT and further centrifuged at 600× *g*. Sperm cells were incubated in 100 µL of Triton X-100 at 0.3% supplemented with the secondary antibody (Goat anti-Mouse IgG (H þ L) Cross-Adsorbed Secondary Antibody, Alexa Fluor 488, Thermo Fisher, Spain) at a final dilution of 0.25 µg/mL. After 30 min of incubation, spermatozoa were rinsed twice by centrifugation in PBS. The final pellet of spermatozoa was diluted in 500 µL of PBS carrying out the analysis immediately by flow cytometry (Supplementary Figure S1d–f). ProAKAP4 staining and specificity was confirmed by fluorescence microscopy (Supplementary Figure S1g). For this assay, eight individual males were analyzed (1 ejaculate per male) including the same males in each experimental group.

Flow cytometry acquisition was performed in a flow cytometer (MACSQuant Analyser 10, Miltenyi Biotech, Madrid, Spain) equipped with three lasers emitting at 405 nm, 488 nm, and 635 nm and 10 photomultiplier tubes (PMTs) (V1 (excitation 405 nm, emission 450/50 nm), V2 (excitation 405 nm, emission 525/50 nm), B1 (excitation 488 nm, emission 525/50 nm), B2 (excitation 488 nm, emission 585/40 nm), B3 (excitation 488 nm, emission 655–730 nm (655LP + split 730), B4 (excitation 499 nm, emission 750 LP), R1 (excitation 635 nm, emission 655–730 nm (655LP + split 730), and R2 (excitation 635 nm, emission filter 750 LP). The system was controlled using MACS Quantify software (Miltenyi Biotech, Madrid, Spain). These excitation and emission wavelengths enabled us to find probe combinations that could simultaneously assess multiple parameters in a large number of spermatozoa (a total of 40,000 events per sample and at least 20,000 sperm cells, at a flow rate of 200–300 cells per second, were acquired) (Supplementary Figure S1a–f). Data were analyzed using FlowJo v.10.2 (Ashland, Wilmington, DE, USA).

### 2.6. RedoxSYS Analysis

Oxidation reduction potential is a measure of the transfer of electrons from a reductant (or antioxidant) to an oxidant and was measured by a galvanostat-based technology, the RedoxSYS assessment (Luoxis Diagnostics, Inc., Englewood, CO, USA). This diagnostic system provides two values: (i) sORP, the integrated balance of oxidants and reductants in a specimen, reported in millivolts (mV); and (ii) cORP, the amount of antioxidant reserves, expressed in microcoulombs (μC). In particular, sperm samples of the three experimental groups (fresh, cooled, and cryopreserved) were washed two times with PBS 1X. After that, 20 μL of sperm samples (1 × 10^6^ sperm cells) were applied to disposable sensors designed by Luoxis, and were inserted into the RedoxSYS diagnostic system, which measured and reported within 4 min the sORP and cORP values. Measures were registered in triplicate for each sample. The average values for sORP and cORP were recorded. Finally, these data were presented as mV/10^6^ sperm for sORP and μC/10^6^ sperm for cORP. A total of five individual males were analyzed (1 ejaculate per male) including the same males in each experimental group.

### 2.7. ProAKAP4 ELISA Assay

Sperm samples of the three experimental groups (fresh, cooled, and cryopreserved) were first washed with PBS. Then 10‒20 million sperm were added to 300 µL of commercial lysis buffer for ELISA quantification using the proAKAP4 ELISA kit (4BioDx, 4VDX-18K7, Lille, France). The standards and samples of different experimental groups were processed in triplicate following the manufacturer’s instructions. Briefly, 50 µL of semen was added to each well of the antibody- precoated plate with the proAKAP4 antibody and incubated for 1 h and 30 min. After washing, the substrate solution was then added to each well and incubated for 30 min. Horseradish peroxidase was employed to detect alkaline phosphatase. Color intensity was proportional to the proAKAP4 concentration in the sperm samples. The color reaction was stopped using a stop solution for 2 min and the absorbance was measured by spectrophotometry at 450 nm (Biotek, Gene 5 Microplate Reader, Winooski, VT, USA). A standard curve was determined for concentrations of proAKAP4 between 8.1 ng/mL and 600 ng/mL. Results of proAKAP4 concentrations in the ram semen were expressed in ng/mL in 1 × 10^6^ spermatozoa. A total of eight individual males were analyzed (1 ejaculate per male) including the same males in each experimental group.

### 2.8. Statistical Analysis

Statistical analysis was carried out using Prism 8 (GraphPad Software, San Diego, CA, USA) and SPSS v. 22 (SPSS Inc, Chicago, IL, USA). Significant differences were considered to have *p* values < 0.05. Data were submitted to Kolmogorov‒Smirnov and Levene’s tests to verify the normality and homogeneity of variances, respectively. Data were analyzed by one-way ANOVA or Kruskal‒Wallis in non-normally distributed data. Results are expressed as the mean ± S.E.M. Pearson and Spearman correlation coefficients were calculated between seminal parameters. The reliability of the scoring systems was tested with the correlation coefficient (R squared). Non-significant differences are represented by: ns. The number of asterisks (*) indicates the significance levels: one asterisk (*) indicates *p* < 0.05, two asterisks (**) indicate *p* < 0.01, three asterisks (***) represent *p* < 0.001 and four asterisks (****) indicate *p* < 0.0001. A principal component analysis (PCA) and correlation matrix including all experimental groups (fresh, cooled and cryopreserved) were performed for the set of sperm quality markers. A total of 5‒8 individual males were analyzed in each experimental group.

## 3. Results

### 3.1. Sperm Motility and Multiparametric Flow Cytometry Analyses

**Figure 1:**
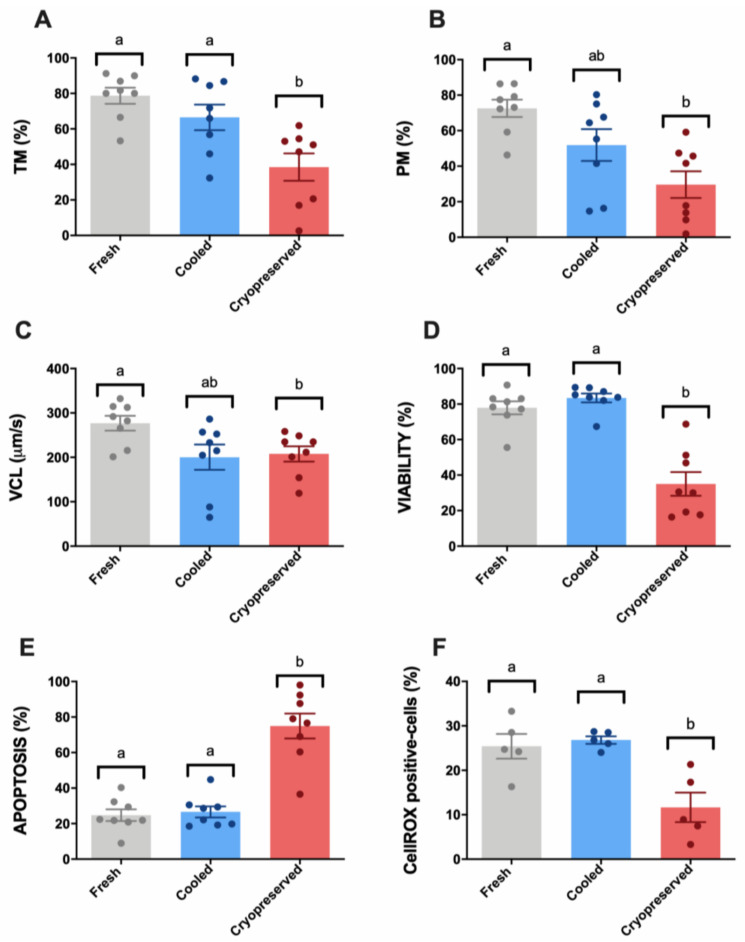
Ram sperm motility and multiparametric flow cytometry analyses in the three experimental groups (fresh, cooled, and cryopreserved samples). (**A**) Total motility (TM, %); (**B**) progressive motility (PM, %); (**C**) curvilinear velocity (VCL, μm/s); (**D**) Zombie negative cells (viability, %); (**E**) caspase 3/7 positive cells (apoptosis, %); (**F**) CellROX-positive cells (sperm ROS levels, %). The same five-eight males were analyzed in each experimental group. Graph dots represent individual male ejaculates. Significant differences (*p* < 0.05) are represented with different letters.

Sperm samples after cryopreservation suffered an important detrimental effect concerning TM and PM parameters compared to the other experimental groups (fresh and cooled) (Figure 1A,B). The cryopreserved samples presented a significantly (*p* < 0.05) altered pattern of sperm movement concerning VCL in comparison with fresh controls (Figure 1C). In contrast, cooled samples did not show significant differences to fresh controls concerning TM, PM, and VCL (Figure 1A–C).

Concerning multiparametric flow cytometry analyses, cryopreserved samples presented a significant (*p* < 0.05) viability decrease compared to fresh and cooled experimental groups (Figure 1D). Non-significant differences were found between fresh and cooled samples in this parameter. For apoptosis occurrence, we found the same pattern: a significant increase (*p* < 0.05) of caspase 3/7 stained cells was observed in cryopreserved samples compared to fresh and cooled ones (Figure 1E). According to these results, CellROX-stained cells, which present high ROS levels, suffered a significant decrease (*p* < 0.05) after thawing (Figure 1F).

**Figure A1:**
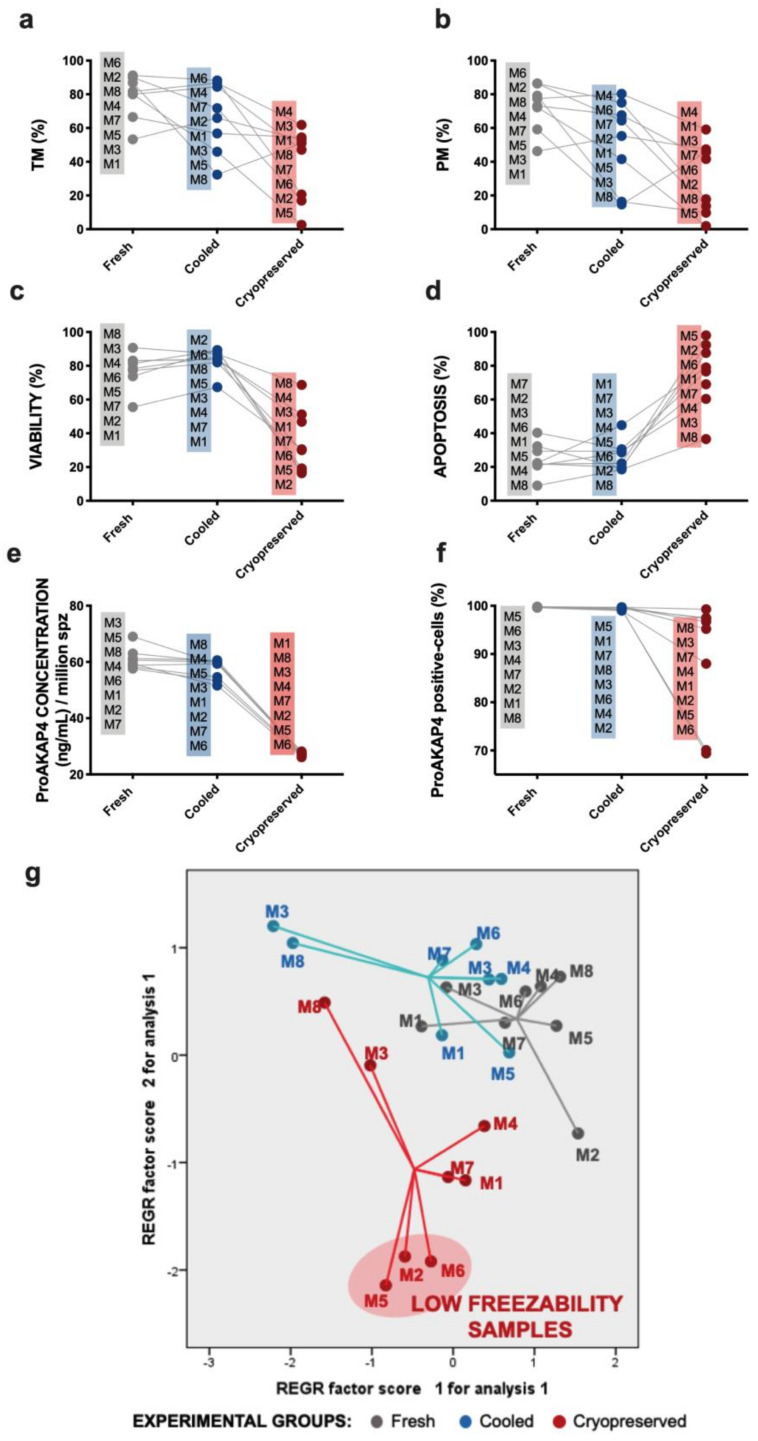
Individual representation and identification of eight rams per experimental group (fresh, cooled, and cryopreserved): motility (**a**), progressive motility (**b**), viability (**c**), apoptosis (**d**), ProAKAP4 concentration (**e**), and ProAKAP4 expression (**f**). (**g**) Scatter plot of males of experimental groups (fresh, cooled, and cryopreserved) separated by the two principal components obtained by principal component analysis (PCA) to reduce the dimensionality of the dataset.

Different subclasses of freezability were observed (Appendix A, Figure A1) among males. Three males (M2, M5, and M6) (Appendix A, Figure A1a–f) presented a reduced cryopreservation tolerance according to sperm motility and multiparametric flow cytometry analyses, confirmed by the different positions in the scatter plot of individual males obtained by a principal component analysis (PCA) considering these parameters (Appendix A, Figure A1g).

**Figure 2:**
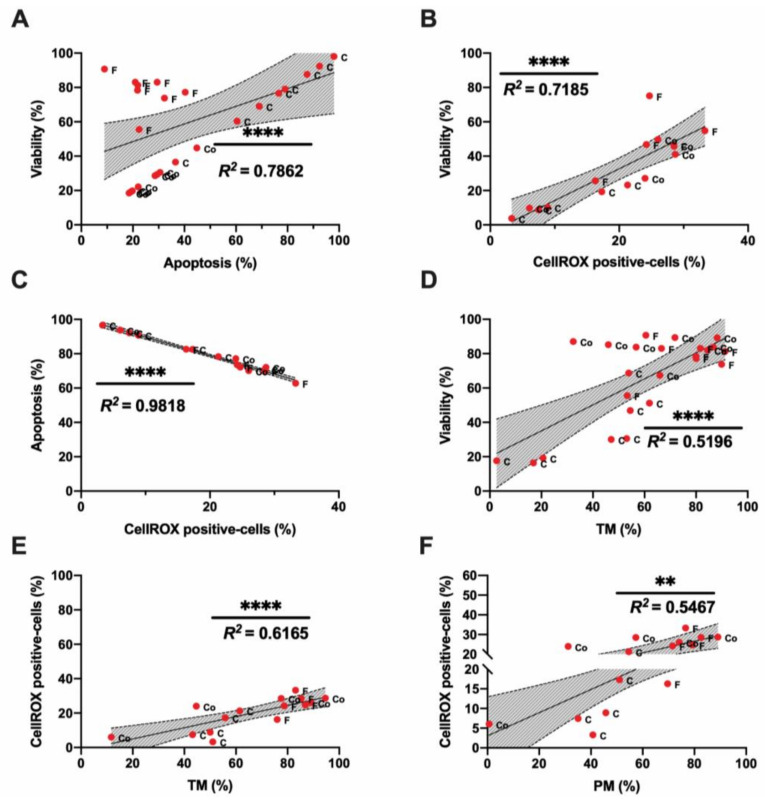
Correlations of ram sperm flow cytometry and motility parameters in the three experimental groups: fresh (F), cooled (Co) and cryopreserved samples (C). (**A**) Correlation between viability (%) and apoptosis (%); (**B**) correlation between viability (%) and CellROX-positive cells (%); (**C**) **c**orrelation between apoptosis (%) and CellROX-positive cells (%); (**D**) correlation between viability (%) and total motility (TM, %); (**E**) Correlation between CellROX-positive cells (%) and total motility (TM, %); (**F**) **c**orrelation between CellROX-positive cells (%) and progressive motility (PM, %). The same five-eight males were analyzed in each experimental group. In correlation studies, a significant correlation is represented by a different number of asterisks; the R squared value is included in each graph. Non-significant correlations are included in the correlation matrix (Appendix A, Figure A2). The number of asterisks (*) indicates the significance levels: two asterisks (**) indicate *p* < 0.01 and four asterisks (****) indicate *p* < 0.0001. Red dots represent all males (five–eight). Each dot is identified by a label with its experimental group (F-fresh, Co- cooled and C-cryopreserved samples).

**Figure A2:**
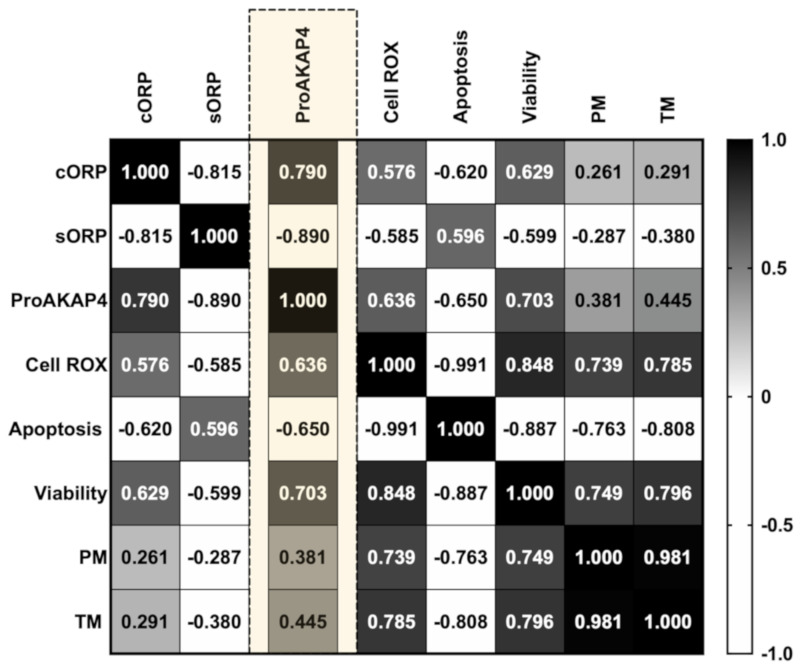
Correlation matrix of all sperm quality markers studied. The three experimental groups (fresh, cooled, and cryopreserved) are included to calculate correlation matrix. The R squared value between two parameters is represented in each cell. Black indicates positive correlations and white indicates negative relationships (not included in the individual correlation studies presented in the manuscript). The color intensity represents the strength of the correlation between two sperm quality parameters. To perform the correlation matrix of sperm quality parameters, the same number of samples is required in each analysis; for this reason, a total of five males were analyzed in each experimental group.

Different correlations among sperm quality parameters were found when we analyzed the motility index compared to sperm viability, apoptosis, and ROS content (Figure 2A–F). The highest correlation among the traditional sperm quality parameters was found between CellROX-positive cells and apoptosis events (Figure 2C). Concerning motility parameters, TM presented the highest correlation with ROS content (Figure 2E). Therefore, the ROS probe showed a significant correlation with sperm motility, viability, and apoptosis. All the correlation studies of sperm quality parameters are included in a correlation matrix in Appendix A (Figure A2).

### 3.2. Novel Sperm Quality Markers: Oxidation Reduction Potential

**Figure 3:**
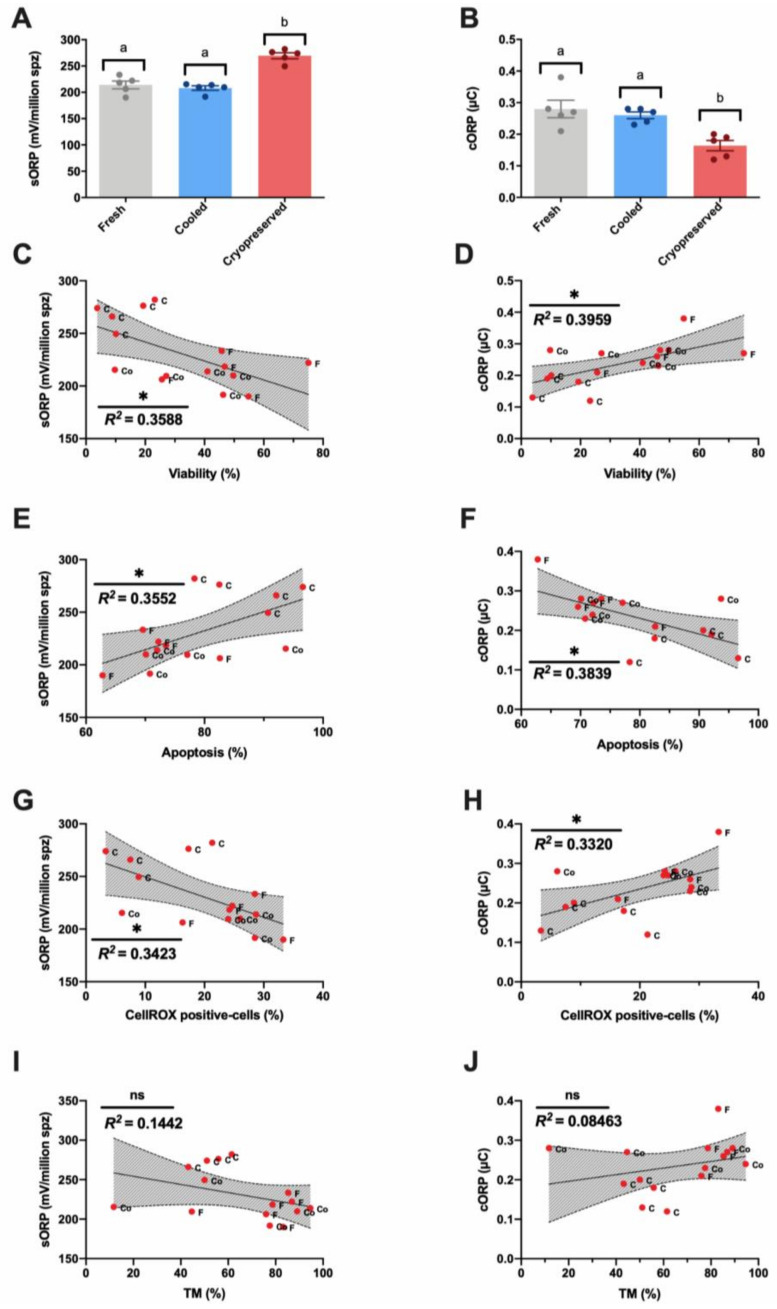
Ram sperm RedoxSYS analyses in the three experimental groups (fresh, cooled, and cryopreserved samples). (**A**) Static ORP (sORP) index (mV/10^6^ sperm); (**B**) capacitance ORP (cORP) index (μC/10^6^ sperm); (**C**,**D**) Correlation between sORP or cORP and viability (%); (**E**,**F**) correlation between sORP or cORP and apoptosis (%); (**G**,**H**) correlation between sORP or cORP and CellROX-positive cells (%); (**I**,**J**) Correlation between sORP or cORP and total motility (TM, %). The same five-eight males were analyzed in each experimental group. In the RedoxSYS analysis, significant differences (*p* < 0.05) are represented with different letters. In correlation studies, the R squared value is included in each graph, a significant correlation is represented by a different number of asterisks. The number of asterisks (*) indicates the significance levels: one asterisk (*) indicates *p* < 0.05 and ns indicates non-significant differences. All correlations are included in the correlation matrix (Appendix A, Figure A2). Red dots represent all males (five–eight). Each dot is identified by a label with its experimental group (F-fresh, Co-cooled and C- cryopreserved samples).

The integrated balance of oxidants and reductants (sORP) showed a significant increase (*p* < 0.05) in cryopreserved samples in contrast to fresh and cooled sperm (Figure 3A). Concerning the capacitance ORP (cORP), and in accordance with the obtained sORP values, cryopreserved samples presented significantly (*p* < 0.05) lower cORP values compared to fresh and cooled experimental groups (Figure 3B). RedoxSYS indexes showed a significant but moderate strength of relationship with viability (Figure 3C,D), apoptosis (Figure 3E,F), and ROS content (Figure 3G,H), given by their respective correlation rates. Moreover, non-significant correlations were found between RedoxSYS indexes (sORP and cORP) and motility (Figure 3I,J). All the correlation studies of sperm quality parameters are included in a correlation matrix in Appendix A (Figure A2).

### 3.3. Novel Sperm Quality Markers: ProAKAP4 Expression and Quantification

**Figure 4:**
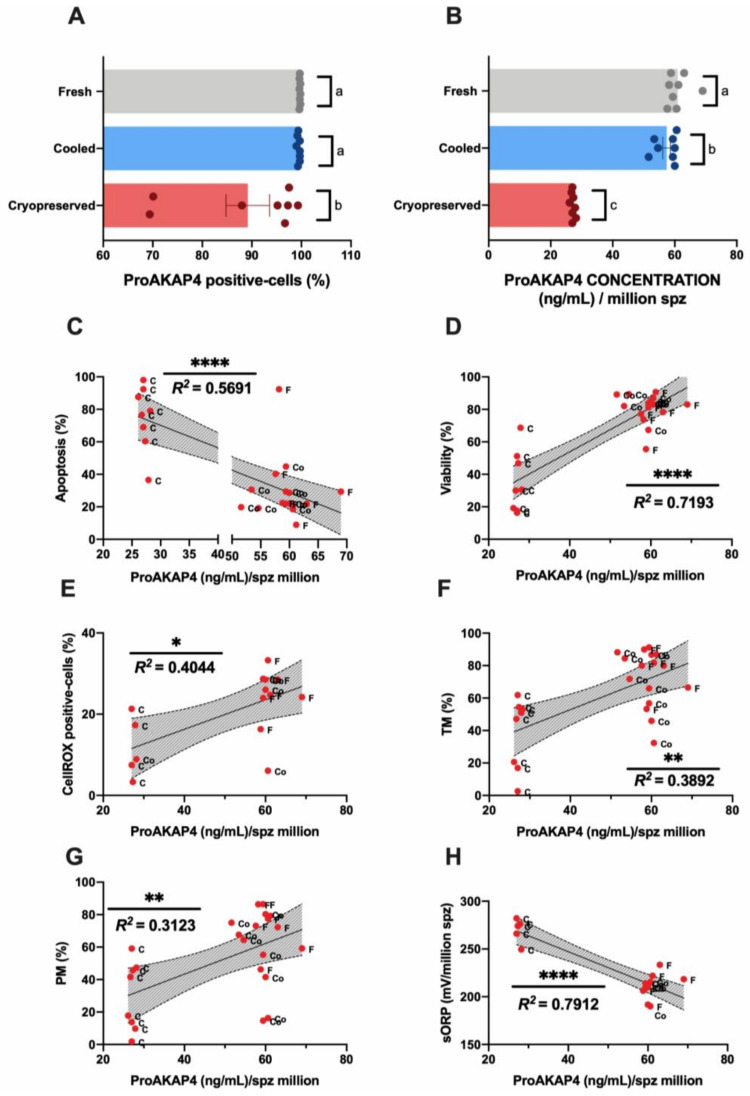
Ram sperm ProAKAP4 analyses in the three experimental groups (fresh, cooled, and cryopreserved samples). (**A**) ProAKAP4-positive sperm population by flow cytometry (%); (**B**) ProAKAP4 concentration (ng/mL) in each experimental group; (**C**) correlation between ProAKAP4 concentration (ng/mL) and apoptosis (%); (**D**) correlation between ProAKAP4 concentration (ng/mL) and viability (%); (**E**) correlation between ProAKAP4 concentration (ng/mL) and CellROX-positive cells (%); (**F**) correlation between ProAKAP4 concentration (ng/mL) and total motility (TM, %); (**G**) correlation between ProAKAP4 concentration (ng/mL) and progressive motility (PM, %); (**H**) correlation between ProAKAP4 concentration (ng/mL) and sORP (mV/10^6^ sperm). The same eight males were analyzed in each experimental group. In ProAKAP4 sperm analysis, significant differences (*p* < 0.05) are represented with different letters. In correlation studies, the R squared value is included in each graph, a significant correlation as represented by a different number of asterisks. The number of asterisks (*) indicates the significance levels: one asterisk (*) indicates *p* < 0.05, two asterisks (**) indicate *p* < 0.01 and four asterisks (****) indicate *p* < 0.0001. Non-significant correlations are included in the correlation matrix (Appendix A, Figure A2).

The sperm population positive for the ProAKAP4 protein was characterized by flow cytometry (Figure 4A). Although in fresh and cooled samples most of the spermatozoa (>95%) expressed ProAKAP4, a significant (*p* < 0.05) decrease was observed after sperm cryopreservation (Figure 4A). Concerning the ProAKAP4 concentration (measured by ELISA) (Figure 4B), we found significant differences in protein levels among the three experimental groups (Figure 4B). Cooled samples kept at 15 °C for 6 h presented a significant reduction in ProAKAP4 concentration compared to fresh samples (Figure 4B). Cooled sperm had higher protein levels than cryopreserved samples (Figure 4B). In addition, ProAKAP4 levels displayed a significant but moderate correlation (*p* < 0.005) with apoptosis (Figure 4C), ROS levels (Figure 4E), and motility parameters (TM and PM) (Figure 4F,G). A high correlation rate given by the R squared value was registered among ProAKAP4 and sperm viability (Figure 4D). The strongest relationship was found between sORP and ProAKAP4 concentration (Figure 4H).

## 4. Discussion

The optimization of short- and long-term sperm conservation methods in rams could improve AI protocols in ovine species to ensure that the semen can colonize and migrate through the cervix [4,5,7,60,61].

In this scenario, preservation methods could induce different types of undetectable damage to stored sperm that could interfere with cervix migration and colonization, with negative consequences for fertility [1,4]. Specifically, in sperm chilled to 5 °C, in vitro assays have revealed that refrigerated ram spermatozoa are able to maintain their viability, motility, and mucus penetration capacity for days, but their fertility in the field decreases long before the deterioration of these parameters becomes apparent [1]. New markers and integrative studies on sperm quality assessment could be more predictive and reinforce the optimization of the current conservation protocols for a better preservation of sperm functionality and fertility. Consequently, we performed multiparametric analyses of ram sperm quality at different levels, including different traditional analyses in combination with new ones, after cooling and freezing protocols compared to fresh controls. We observed that, in the first level of analysis carried out by SCA, cryopreserved samples exhibited detrimental effects on their motility and kinetic parameters (Figure 1). Concerning cooled samples, although some significant differences (*p* < 0.05) were found in the sperm’s movement pattern, when we analyzed sperm motility (TM and PM), we did not find any differences between fresh and cooled samples that could explain the observed decrease in fertilization ability (Figure 1A–C). This result is in accordance with previous research works that claimed that chilled sperm is able to maintain viability and motility, but fertility decreases long before these sperm quality parameters are affected [1].

In the second level of assays, a multiparametric flow cytometry analysis was performed, revealing the same pattern observed in SCA parameters (Figure 1D,E). Cryopreserved samples suffered a significantly negative effect on their viability and ROS content, triggering apoptosis (Figure 1). This significant detrimental effect was not present in cooled samples, which maintained these parameters compared to fresh samples (Figure 1D,E). In addition, ROS content showed a strong correlation with sperm viability, apoptosis, and motility (Figure 2B,C,E). At this point, we suggest that many factors affect sperm physiology at different levels (cellular and molecular) during storage that could remain undetectable with traditional sperm quality analysis methods. In this sense, one of the main consequences of semen storage is the oxidative stress produced not only by the production of free radicals and the accumulation of waste substances, but also by a decrease in the antioxidant capacity of the sample [62,63,64]. Specifically, the tests currently available mainly focused on ROS measured by chemiluminescence, total antioxidant capacity (TAC), and malondialdehyde (MDA) assay for lipid peroxidation detection [31,65]. However, these traditional tests only provide a single dimension of oxidative stress, quantifying either ROS or antioxidants [65]. It is necessary to establish novel technologies that include all of the constituents of oxidative stress in an integrative manner to provide a better understanding of the true redox state. In response to these requirements, novel advancements based on redox balance have emerged and have been successfully applied in different mammal species including humans and stallions [31,64,66]. The measuring of oxidation‒reduction potential (ORP) is a direct value of oxidative stress that includes the relative proportions of oxidants (ROS) to reductants (antioxidants) [31,64]. RedoxSYS provides a global analysis of oxidative stress (ROS levels and antioxidant capacity); this technology could represent an important step in ram sperm evaluation after cooling or freezing protocols [37,38,39,40]. For this reason, we decided to include this novel assay in our study. Surprisingly, cooled and fresh samples presented similar sORP and cORP values that differed significantly from the cryopreserved samples (Figure 3A,B). In our case, the ROS levels detected by CellROX (Figure 1F) and RedoxSYS analyses (Figure 3A,B) shown the same pattern among the experimental groups, registering a significant detrimental effect (*p* < 0.05) only in cryopreserved samples. As a consequence of this, a moderate correlation was confirmed among RedoxSYS index and CellROX-positive cells (Figure 3G,H). The significant decrease obtained in CellROX positive-cells after cryopreservation could be explained according to previous results obtained by other authors [67]. In these studies, the authors evidenced that the electron transfer blocking (that could be trigger by cryopreservation) provoked the interruption in superoxide anion production [67]. Considering that the CellROX probe mainly detects superoxide anion according to manufacture description, the increase in this anion may reflect intense mitochondrial activity rather than oxidative stress [67].

In addition, molecular markers based on protein characterization could reinforce this novel deep level of analysis. These biomolecules could provide new insights into the mechanisms underlying sperm cryodamage and could act as freezability biomarkers as well as for the development of new strategies to improve sperm cryopreservation outcomes in ruminants [45]. Novel proteins such as AKAP4 and ProAKAP4 have recently taken on great relevance in the sperm quality assessment field as predictors of fertilization success in different mammal species [46,47,68] due to their close correlation with sperm motility. Moreover, taking into account the recently discovered relationship with oxidative stress [50], these proteins could be suitable candidates to incorporate in new sperm quality analyses in rams. When we incorporated new markers such as ProAKAP4 in a third level of analysis, cryopreserved samples showed the same pattern observed in the other parameters, with a negative effect on ProAKAP4 expression (Figure 4A) and total concentration (Figure 4B). Moreover, the protein expression and concentration suffered the sharpest decrease in three studied males (M2, M5, and M6; Figure 4A, Appendix A, Figure A1e,f) that presented poor freezability compared to the others. This low cryopreservation tolerance of samples was confirmed by a principal component analysis (PCA), including sperm motility and flow cytometry multiparametric analyses (Appendix A, Figure A1g) to reduce the data dimensionality. The cryopreserved samples were well separated, with the second two principal components as two new freezability subclasses (Appendix A, Figure A1g). These results confirmed the suitability and accuracy of this protein as a molecular marker of sperm quality after ram sperm cryopreservation.

Although the proportion of sperm expressing ProAKAP4 remained unaltered between fresh and cooled samples (Figure 4A), the amount of this protein between these experimental groups was significantly (*p* < 0.05) different (Figure 4B). Cooled samples showed lower values than control ones (*p* < 0.05). Moreover, we observed a more homogeneous pattern in ProAKAP4 levels as a response to the same protocol of cooling and freezing among individual males (Appendix A, Figure A1e,f) compared to sperm motility parameters (TM and PM) (Appendix A, Figure A1a,b). This reinforces the use of the ProAKAP4 value as an acute and robust biomarker in this type of protocols. Surprisingly, when we analyzed the correlation of sperm quality parameters with this novel marker, the highest correlation rates were registered for ProAKAP4, viability and sORP (Figure 4D,H), confirmed by the very close distribution in the component plot graph of the principal component analysis to reduce the dimensionality of the dataset (Appendix A, Figure A1g). Although ProAKAP4 has been widely correlated with sperm motility traits by different authors [41,42,43,44,45], the protein concentration has never been correlated with several sperm quality parameters such as viability or sORP.

However, despite these existing correlations, an alteration in ProAKAP4 levels in cooled samples is not accompanied by a modification of RedoxSYS or viability parameters in these samples, as we can observe in our results. This could be explained by taking into account the recent studies performed on human sperm that demonstrated that low levels of ROS cause functional lesions in the mature spermatozoon, specifically in structural proteins such as chaperone HSPA2, which compromises their fertilization potential, but does not negatively impact their viability [69]. In our case, the low levels of oxidative stress that cooled samples suffer could have the ability to disrupt the sperm surface architecture and its related scaffold proteins (such as AKAP4), representing the first sign of sperm damage [47,48]. The decrease in ProAKAP4 protein concentration has previously been related to an impairment in the process of sperm capacitation, which confers upon spermatozoa the ability to participate in sperm‒egg recognition and could explain the decrease in fertility [69,70].

**Figure A3:**
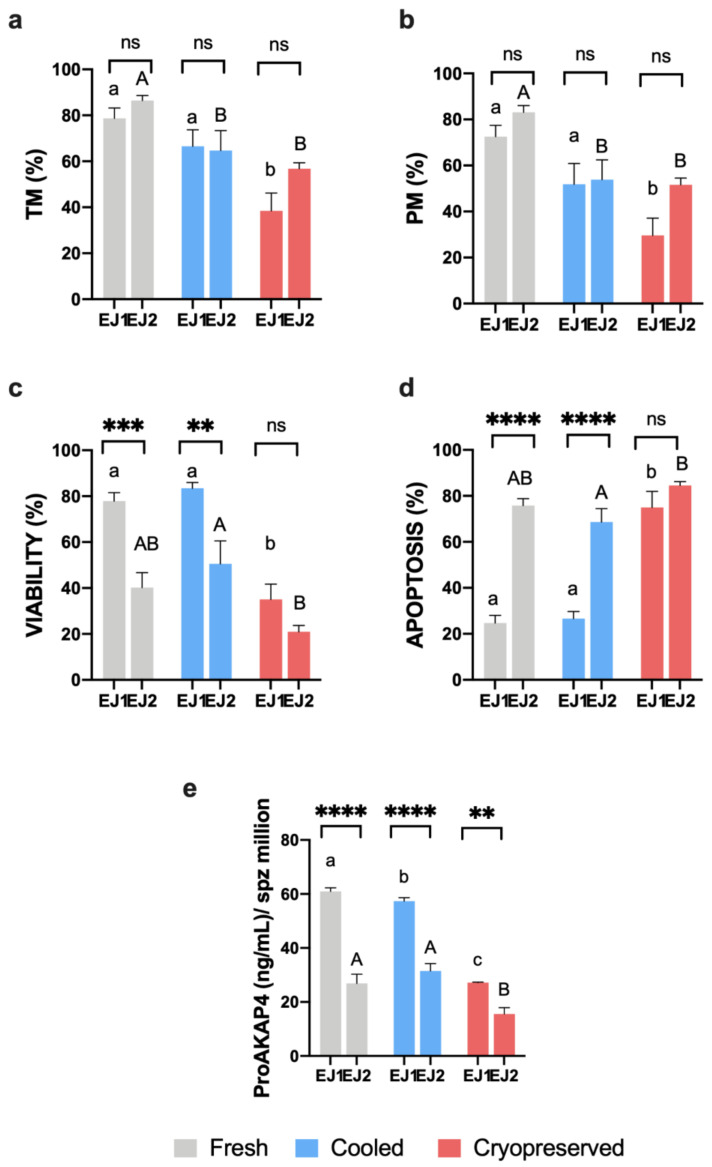
(**a**,**b**) Ram sperm motility (TM and PM, %), (**c**) viability (%), (**d**) apoptosis (%) and (**e**) ProAKAP4 concentration (ng/mL) in the three experimental groups (fresh, cooled, and cryopreserved samples) in a different ejaculate (EJ2) of some analyzed males comparing to the first ejaculate obtained (EJ1). EJ1 and EJ2 were obtained in a different season. A total of six males were analyzed considering the same males in each experimental group. Significant differences (*p* < 0.05) between ejaculates in each experimental group are represented with asterisks: two asterisks (**) indicate *p* < 0.01, three asterisks (***) represent *p* < 0.001 and four asterisks (****) indicate *p* < 0.0001. Significant differences among experimental groups in EJ1 are represented with different lowercase letters. Significant differences among experimental groups in EJ2 are represented with different capital letters.

Finally, to corroborate ProAKAP4 biomarker robustness and to check its relationship with viability rather than motility tests, we analyzed a different ejaculate (EJ2) of some of the analyzed males. This EJ2 was obtained in a different season (out of breeding season) using the same experimental groups (fresh, cooled, and cryopreserved) and comparing the obtained results to the first ejaculates (breeding season) employed in this study (Appendix A, Figure A3). Concerning motility parameters, non-significant differences were found between EJ1 and EJ2 (Appendix A, Figure A3a,b), however, motility decreased significantly (*p* < 0.05) in cooled samples compared to the fresh ones in contrast to the obtained results in EJ1. When we observed viability and apoptosis results (Appendix A, Figure A3c,d), EJ2 suffered a significant (*p* < 0.05) detrimental effect in fresh and cooled samples comparing to EJ1. This different sperm quality between EJ1 and EJ2 evidenced by viability and apoptosis was accompanied by a significant (*p* < 0.05) decrease in ProAKAP4 concentration in all experimental groups (Appendix A, Figure A3e) evidencing its high correlation with viability and apoptosis and its accuracy and robustness as sperm quality marker in ram.

## 5. Conclusions

It is important to highlight that ProAKAP4 concentration could be a more accurate fertility predictor than other ram sperm quality parameters. This work allows us to confirm the role of this protein as a sperm biomarker in ram and could be applied to improve AI technology in this species through the best diagnostic of sperm quality allowing us to optimize conservation (freezing and cooling) protocols.

## Supplementary Materials

The following are available online at https://www.mdpi.com/2218-273X/10/7/1046/s1, Figure S1: Flow cytometry analyses and ProAKAP4 localization.
