# Supplementary figures and images for: ProAKAP4 as Novel Molecular Marker of Sperm Quality in Ram: An Integrative Study in Fresh, Cooled and Cryopreserved Sperm
Source: Biomolecules. 2020 Jul 14;10(7):1046. doi: 10.3390/biom10071046 (PMC7408074; doi:10.3390/biom10071046)

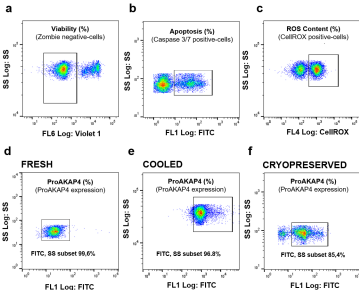

**g**

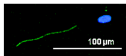

Supplement: Supplementary file 1 [file biomolecules-10-01046-s001.pdf]
